# High similarity of IgG antibody profiles in blood and saliva opens opportunities for saliva based serology

**DOI:** 10.1371/journal.pone.0218456

**Published:** 2019-06-20

**Authors:** Peter Hettegger, Jasmin Huber, Katharina Paßecker, Regina Soldo, Ulrike Kegler, Christa Nöhammer, Andreas Weinhäusel

**Affiliations:** Austrian Institute of Technology, Center for Health and Bioresources, Molecular Diagnostics, Vienna, Austria; University of Pisa, ITALY

## Abstract

Saliva based diagnostics is a rapidly evolving field due to the large diagnostic potential and simple sample collection. Currently only few individual molecules were investigated for their diagnostic capabilities in saliva. A systematic comparison of IgG antibody profiles in saliva and plasma is still missing in scientific literature. Our hypothesis is that IgG profiles in plasma and saliva are highly similar for each individual. As a consequence, one could implement practically any plasma based IgG assay (classical serology) as saliva based assay. In other words, the IgG antibodies found in blood are also accessible from saliva. We confirm our hypothesis by comparing IgG reactivities towards protein and peptide antigens. We isolated saliva IgG with high purity and demonstrate that plasma IgG reactivities (classical serology) can be inferred from saliva. As a showcase we perform Hepatitis B virus antibody (plasma-)titer determination from saliva. Additionally we show that plasma and saliva IgG profiles of 20 individuals are highly similar for 256 peptide antigens and match (unsupervised) with high probabilities. Finally, we argue for generalisation to the complete IgG antibody profile. The presented findings could contribute greatly to the development of saliva based diagnostic methods of numerous antibody based tests.

## Introduction

The increasing expenses of healthcare systems and the increasing number of patients require an increase in efficiency in all healthcare related processes. A promising opportunity to increase efficiency in diagnostic, screening or monitoring applications is the usage of saliva as diagnostic fluid due to good sample accessibility and a large variety of potential diagnostic and monitoring possibilities [1–10]. Diagnosis of diseases and screening e.g. for epidemics is frequently done stationary by physical examination and by performing blood tests. Using saliva instead of blood has potential benefits especially for screening a high number of people or for usage in decentralized regions like for point of care (POC) or home testing: (i) sampling of saliva is simple, does not necessarily need trained staff and is usually readily available; (ii) saliva collection does not need health care professionals for drawing blood and in principle not even sterile goods as in case of blood sampling—for many applications saliva could be microbiologically stabilized after collection; (iii) collection can be done anywhere and processing can be done centrally if the analyte of interest can be stabilized; (iv) high numbers of samples can be collected easily. However, several factors cause saliva to be a relatively heterogenic body fluid among individuals as compared to e.g. blood or plasma. These factors include: (i) age related and genetically determined effects which cause differences in viscosity or relative water, protein (e.g. mucins, enzymes and antibodies) as well as ion content; (ii) smoking as this could cause high particle load and discoloration of saliva; (iii) differences in salivation; (iv) microbiome in the oral cavity; (v) disease associated alterations like increased blood content due to bleedings in the oral cavity or associated structures and many more. These differences could be challenging for sample collection and preparation and potentially interfere with subsequent analyses [11]. The saliva composition can additionally be altered by the collection procedure or any form of stimulation like e.g. using chewing gums, by psychological stimulation or the usage of extraction solutions [12,13]. Thus, accurately defined sampling procedures and circumstances are essential for reproducible analyses. Furthermore, many analytes of interest are found in much lower concentration as compared to blood which additionally challenges the analysis procedures and methods [5].

Antibodies isolated from blood or saliva could be potentially used for diagnosis and monitoring of diseases and medical conditions related to or associated with (IgG) antibodies in humans. Numerous potential applications have been described for various diseases like: autoimmune disorders (multiple sclerosis [14], inflammatory bowel disease [15], systemic lupus erythematosus (SLE) [16]), viruses (HIV [17–19], dengue [20], measles, mumps & rubella [21]), antibodies against tumour associated antigens [22–25] and parasites [26], to name just a few. Some of these have already been shown to be detectable and (in principle) diagnostically utilizable in saliva [3,18–21] and to date one saliva based HIV test is already on the market [18]. However, all of the mentioned studies investigated only specific, disease related antibody idiotypes or sets of idiotypes. A more general approach, detached from a specific application or medical condition, is still missing in scientific literature.

Our hypothesis is that IgG profiles in plasma and saliva are highly similar for each individual. The immunological profile of an individual is for now formally defined as the reactivity of IgGs of that individual against a certain set of antigens. It can hence be seen as an immunological fingerprint for a certain set of antigens. For the sake of readability, a more detailed consideration of the immunological profile as a concept is provided in the Discussion part. By showing high similarity of the immunological profiles in plasma and saliva, we show that the diagnostic potential of saliva is not limited to diseases localized in the oral cavity or anatomically and physiologically associated structures, but is extended to the whole body or, more specifically, at least to any IgG antibody which can be found in blood. So the IgG antibodies found in blood are also accessible from saliva, this is our main result. In the present work we do not focus on the development of a particular diagnostic application for saliva, but we aim at showing that it is in principle possible to develop/adopt a diagnostic test which is based on human plasma IgGs also for saliva. The document is structured in the following way: We show that (i) we can isolate saliva IgG with high purity; (ii) we are able to infer anti-HBV-antibody (plasma-)titer from saliva; (iii) relative IgG antibody reactivities are highly similar in saliva and plasma and we can thus infer plasma IgG reactivities from saliva; (iv) immunological profiles are highly similar in saliva and plasma. Note that (ii)–(iv) are consecutive steps of generalisation. Due to the nature of the immune system it is inherently only possible to observe (iii) and (iv) on a subset of the immunological profile. The validity of generalisation from a subset to the whole immunological profile of an individual is argued in the Discussion part. We thus provide a general framework and present data for showing high similarity (or effectively equality) of the immunological IgG antibody repertoire in plasma and saliva.

## Results

### High purity IgG isolation from saliva

**Fig 1:**
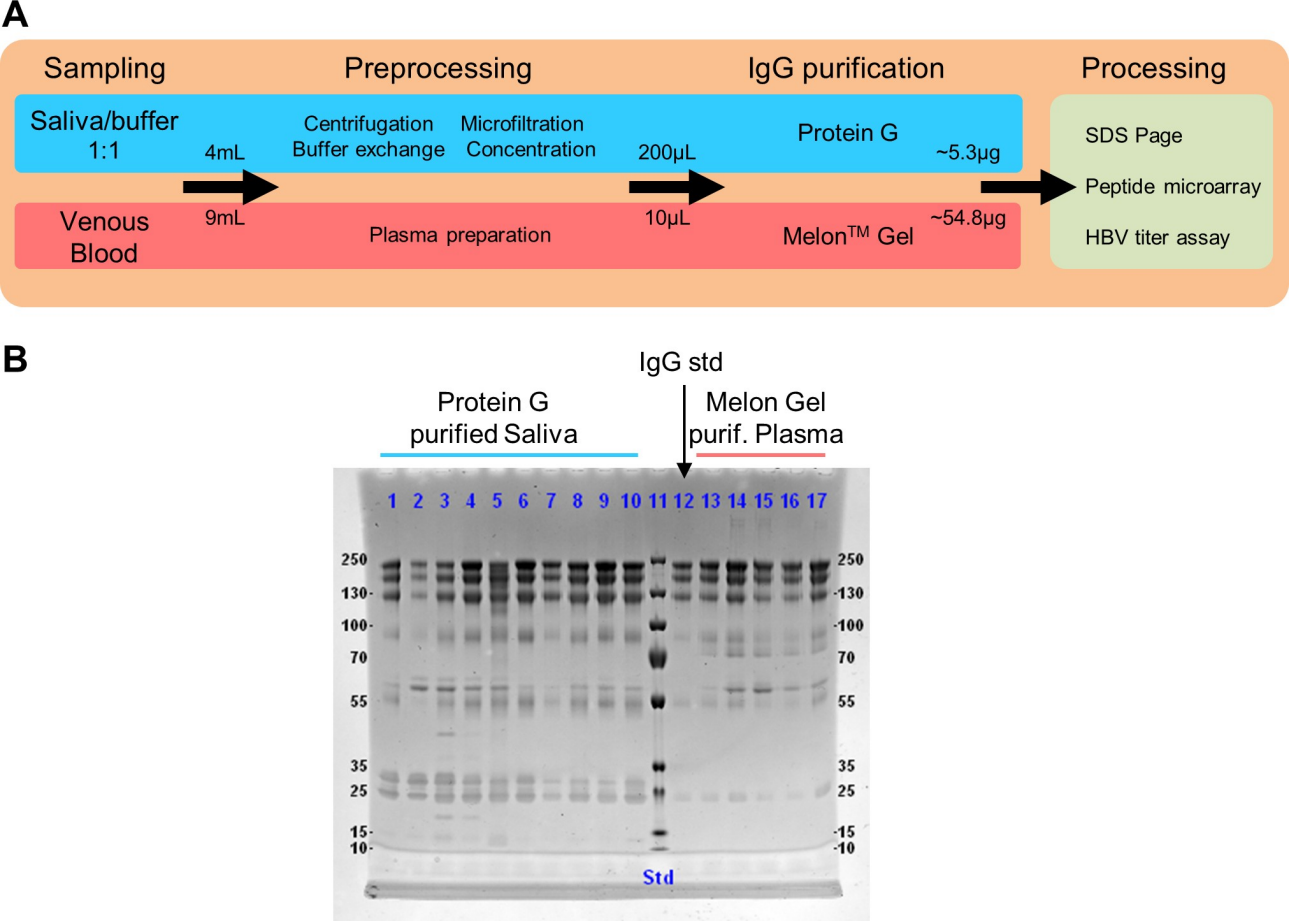
Schematic isolation protocol and purity of IgG isolation from human saliva and plasma samples. (A) Saliva was collected using a commercial saliva collection kit containing citrate buffer. Saliva/buffer mixture was then centrifuged and microfiltrated for removal of heavy components, particles and large molecules. A buffer exchange step with binding buffer was performed for subsequent affinity chromatography using Protein G resin. Plasma was prepared from venous blood and IgG was purified using a commercial purification kit. Saliva and plasma IgG were processed on different assay for subsequent analyses. (B) Non-reducing SDS page of saliva and plasma purified samples. Saliva and plasma isolates from 10 and 5 individuals are shown, respectively. Additionally, a commercial IgG standard is shown (lane 12) for comparative purposes. The most prominent bands are highly similar for all lanes. IgG bands are prominent between 100 and 250kDa marker positions. Average purity of saliva IgG isolates was 79% (± 13% SD) as determined by relative area under the curve (AUC) in the chromatograms (S1 Fig). IgA amount was below LOD of 1μg/mL (total protein content of isolated samples > 100μg/mL) as determined by a calibrated IgA Luminex assay.

We developed a method for isolating IgG from human saliva with high purity as the most important prerequisite for subsequent analyses. In short, saliva was centrifuged for removing heavy components, microfiltrated for removing particles and large molecules and subsequently buffer exchanged with binding buffer for purification by Protein G affinity chromatography (Fig 1A). For comparison of purity and yield, we prepared plasma from venous blood and isolated plasma IgG using a commercial Melon Gel isolation kit. Additionally, a commercial IgG standard was used. Average IgG yield was 5.3μg (range 1.7–12.5 μg) from 2mL saliva and 54.8μg (range 40–76.45μg) from 15μL plasma (S1 Table). Plasma and saliva IgG was further processed with different assays for subsequent analyses. The SDS page for saliva and plasma isolates is shown in Fig 1B. Average saliva IgG purity was 79% ± 13% standard deviation (SD) (S1 Fig) and final IgA concentration was below limit of detection (LOD) of 1μg/mL based on Luminex IgA assays (with total sample protein concentration of more than 50μg/mL). All lanes, including the commercial IgG standard, show comparably prominent IgG bands between 100 and 250kDa marker positions. Saliva purified IgG samples showed hence reasonably high purity for further processing. Comparison of concentrated and microfiltrated saliva with purified IgG is shown in S2 Fig.

### HBV antibody titer determination from saliva

**Fig 2:**
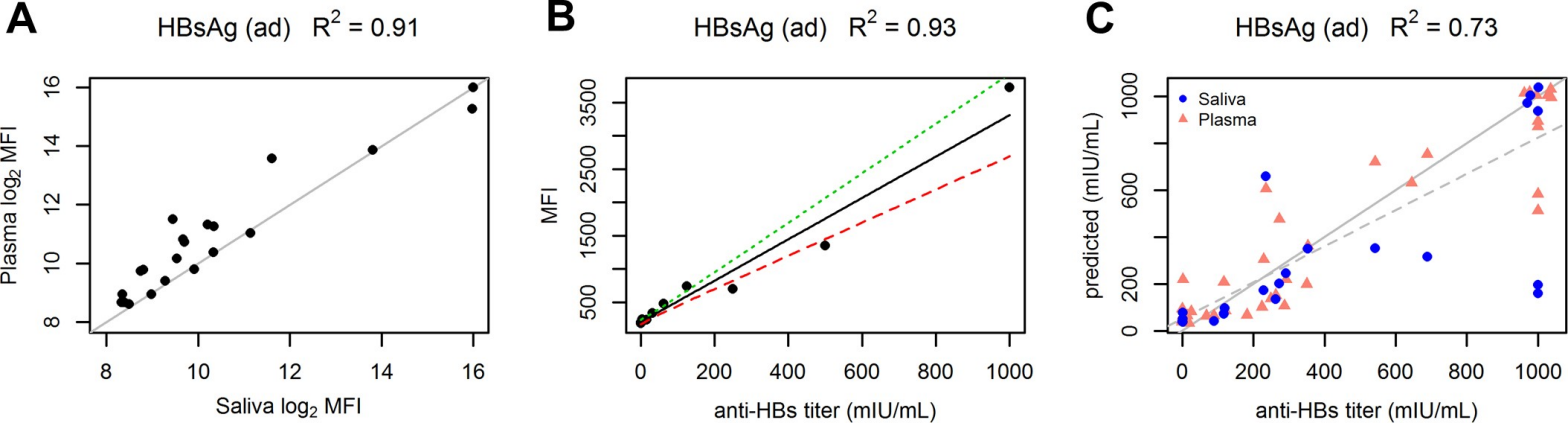
HBV antibody titer determination in plasma and saliva. **Hepatitis B surface antigen subtype ad (HBsAg ad) was used as antigen on self-printed microarrays for titer determination in an indirect immunoassay format.** (A) Plasma and saliva signal intensities for 21 paired human samples. The identity line is shown in grey. (B) Assay calibration with anti-HBs antibody standard. The linear regression line is shown in solid black. Prediction bounds are indicated with red and green dashed lines. (C) Plasma and saliva titer values as determined by the reference routine diagnostic lab (abscissa) and titer predictions from purified plasma IgG and purified saliva IgG as determined with our assay (ordinate). R^2^ of the linear regression is 0.73 (n = 59). The solid line shows the identity line, the dashed line shows the regression fit. Small jitter was added (in the graph, not the data) to (1000,1000) points in C for better illustration (to prevent points lying on top of each other). Paired plasma and saliva samples were available for 21 individuals. Additional plasma samples were available for 17 individuals. Data for all 3 graphs can be found in S2 and S3 Tables. R^2^ is Coefficient of determination (squared Pearson’s r).

As a first proof of principle for showing the equality of plasma and saliva IgG reactivity, we show similarity for a well characterised anti-*Hepatitis B virus* (anti-HBV) antibody which is reactive against a HBV surface antigen. This serves as an illustration for practical feasibility of a saliva IgG based diagnostic routine, as the developed system is suitable for inferring anti-HBV antibody titer (plasma titer) from saliva. Although there are potentially other applications of higher relevance in industry and academia, we have still chosen HBV antibody titer determination as a pilot example as there is generally a high HBV vaccination coverage rate in Austria and we could thus expect positive signals with reasonable variance in our study cohort (n = 38). We want to emphasize, that this should serve as an illustrative example of one possible clinical application. Still, the main focus of the paper is not to develop a specific test which can be used in routine diagnostics, but to show that the IgGs found in blood are accessible from saliva, independent of certain specificities (idiotypes). For the following analyses, we used customized self-printed protein microarrays with recombinant Hepatitis B surface antigen subtype ad (HBsAg ad) for quantification of anti-HBV antibody reactivities. Signal intensities measured in plasma and saliva show high concordance for all 21 individuals (R^2^ = 0.91) for which paired saliva and plasma samples were available (Fig 2A). The assay was calibrated using an anti-Hepatitis-B surface antigen (Anti-HBs) antibody standard from the National Institute for Biological Standards and Control (NIBSC). Coefficient of determination (COD) for the calibration is R^2^ = 0.93 (Fig 2B). For comparison with the antibody titer predicted from our assay, the antibody titer was determined for all individuals from their respective plasma samples by an external routine medical diagnostic lab. This plasma titer was used as a reference for saliva and plasma samples. In contrast, we used our protein-microarray based assay for prediction of the antibody titer values from purified plasma IgG and purified saliva IgG. COD between reference and predicted titer values is R^2^ = 0.73 (Fig 2C). Please note that for 7 out of 59 samples (12%), which are far off the diagonal line, there is a relatively high disagreement between values from our assay and the reference lab. This is not contradictory to the previous claim that we can infer IgG reactivity in plasma from saliva IgG. The disagreement is seen in predicted titer values (Fig 2C), but not in measured signal intensities of plasma and saliva (Fig 2A) and thus is considered a systematic error in titer determination. The differences in titer determination arise presumably as (i) the reference lab uses a mixture of *HBsAg ad* and *HBsAg ay* subtypes; (ii) we use purified IgG, in contrast to whole plasma as used by the reference lab (routine medical diagnostic labs commonly use whole plasma for titer determination). This might result in different matrix effects; (iii) our assay structure (direct immunoassay with fluorescent detection) is different from the assay used by the reference lab (sandwich electro-chemiluminescence immunoassay), see Materials and Methods part.

### IgG antibodies show quantitatively equal reactivity in saliva and plasma

In the following we show that, additionally to the relative reactivities against the HBsAg protein antigen (shown above), also the relative reactivities against peptide antigens (15 amino acids) show high similarity for saliva and plasma samples of all tested individuals. This serves as a first generalisation step from one HBV antigen to multiple antigens of the immunological profile. Paired saliva and plasma samples were taken from 20 individuals and IgG was isolated (Fig 1). Isolated IgG was assayed on peptide microarrays with 158 linear epitopes of *Epstein-Barr Virus* (EBV) and 98 of *Hepatitis B virus* (HBV) antigens. We used EBV and HBV peptide microarrays due to the expected high reactivity resulting from high prevalence (EBV) and high vaccination coverage rate (HBV) and the good reproducibility due to chemical synthesis of the peptides and commercial availability.

**Fig 3:**
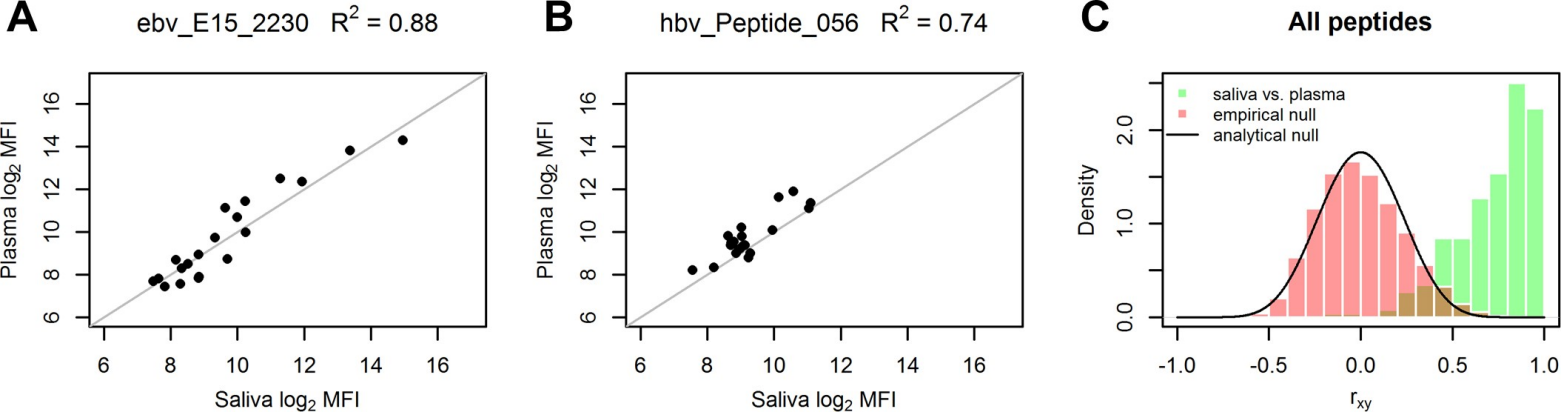
High accordance of IgG reactivities in paired plasma and saliva samples against individual peptides. (A, B) Plasma and saliva reactivities of representative EBV and HBV derived peptides. The identity line is shown in grey. (C) Distributions of Pearson correlation coefficients (r_xy_) between plasma and saliva intensities (green bars) for all 256 peptides (158 EBV and 98 HBV peptides) and null-distributions (red bars and black line, respectively) for comparison. Pearson’s r is shown instead of R^2^ in C for reasons of clarity. 47% of all peptides have r_xy_ > 0.8, 83% have BH FDR < 0.01 (analytical Null). For comparison of resulting correlation coefficients, the null distribution (expected distribution of r in case of zero correlation or random data) is calculated in two ways. The empirical null distribution is generated by random resampling from array data. The analytical null distribution is shown for the case of independent normal variables. Note: the slight positive skew of the empirical null distribution originates from the positive skew of the data (log2 MFI of samples), which is typical for microarray data. R^2^ is Coefficient of determination (squared Pearson’s r).

Reactive peptides show a linear relationship and high accordance of signal intensities between saliva and plasma samples (Fig 3A and 3B). Almost half of the peptides (47%) show Pearson’s r greater than 0.8 or R^2^ > 0.64 for signal intensities in saliva and plasma. All other peptides, except for 2 out of 256, show relatively high r > 0 as compared to the null distributions (Fig 3C). The peptides with low correlation values generally show low mean values and/or low variance of fluorescence intensities indicating low or unspecific reactivity of the according peptides (S3 Fig). Moreover, mean signal intensities and intensity ranges (max minus min) are highly similar in plasma and saliva samples for all peptides (S4 Fig). This indicates, additionally to relative intensities, the high similarity of absolute intensities and hence relative quantities (fraction of antibodies relative to all antibodies in the according body fluid). The assays were normalized by using the same amount of IgG for saliva and plasma arrays and by median normalization between arrays.

Taken together, this data indicates that the IgG antibodies show quantitatively equal reactivity in saliva and plasma arrays. Equivalently, this indicates the possibility of inferring absolute reactivities of plasma IgG species using saliva IgG samples.

### High similarity of saliva and plasma IgG profiles

**Fig 4:**
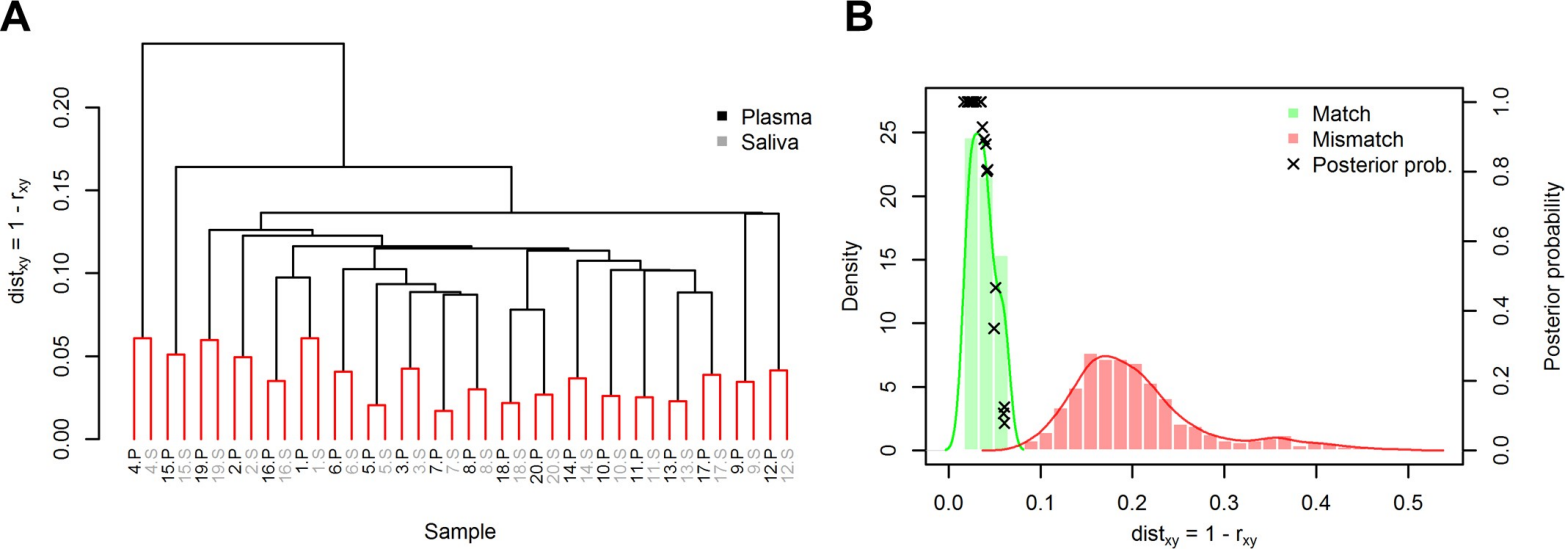
High similarity of IgG profiles between saliva and plasma sample of each individual. (A) Hierarchical clustering of saliva and plasma IgG samples of 20 Individuals. S and P mark the respective saliva and plasma IgG samples for each individual. The samples cluster in a pairwise manner indicating high similarity (dist_match_ < 0.06) of each individual’s saliva and plasma IgG. Single linkage was used and terminal edges are colored red. (B) Histograms of pairwise distances between all samples. The match distances are pairwise distances of intra-individual saliva and plasma (i.e. red pairs in A). Mismatch distances are all other pairwise distances (inter-individual). Note that the match and mismatch histograms do not overlap (dist_mismatch_ > 0.08). Mean match distance is 0.037, mean mismatch distance is 0.205. Kernel density estimates are shown as solid lines. Additionally, estimated posterior probabilities of match P(match|dist_xy_) with prior probability P(match) = 1/1000 are shown for all 20 samples (see text for interpretation of the 1/1000 prior). For each individual, dist_xy_ values of that individual were withheld from estimation of conditional distributions to avoid overestimation of posterior probabilites. Distance function: dist_xy_ = 1 –r_xy_, r_xy_ is Pearson’s r.

We continued comparing plasma and saliva IgG profiles of the 20 individuals on the 256 peptide antigens (see EBV and HBV peptide arrays above). Hierarchical clustering results in pairwise clustering of saliva and plasma IgG samples for all 20 individuals (Fig 4A). This indicates that each individual’s saliva and plasma IgG profiles are more similar to each other than to any other plasma or saliva sample (S5 and S6 Figs). In other words, one could say that the IgG fingerprint is identical in saliva and plasma. Furthermore, the high intra-individual similarity of IgG profiles remains even for samples (same person, same body fluid) taken repeatedly at different days more than 6 weeks separated from initial sampling (S7 Fig and S4 Table).

### Saliva and plasma IgG profiles match with high probability

Looking at all pairwise distances between samples relieves that the match (intra-individual) and mismatch (inter-individual) distances do not overlap in the histograms (Fig 4B). Based on the microarray-data from each sample, one could derive the probability that a specific pair of plasma and saliva samples comes from the same individual. We derive these probabilities in order to emphasize the high accordance of plasma and saliva IgG profiles by means of a hypothetical example from forensics.

In the following, an estimation of (posterior) matching probabilities between plasma and saliva samples of each individual is derived. The conditional probability distributions of match and mismatch distances, *P*(*dist_xy_*|*match*) and *P*(*dist_xy_*|*mismatch*), for two IgG profiles are estimated using kernel density estimation from the pairwise distances of the 20 individuals. Match hereby indicates the probability that the samples come from one individual, mismatch indicates that the samples come from different individuals. Using Bayes’ theorem the posterior matching probability (probability of match given a certain distance value) for each individual’s saliva and plasma IgG profiles is

$$P\left(match|dist_{xy}\right)=\frac{P\left(dist_{xy}|match\right)P\left(match\right)}{P\left(dist_{xy}\right)}$$

with the conditional probability *P*(*dist_xy_*|*match*), the prior probability *P*(*match*) and the marginal probability *P*(*dist_xy_*) = *P*(*dist_xy_*|*match*)*P*(*match*)+*P*(*dist_xy_*|*mismatch*)(1−*P*(*mismatch*)). For the calculation of the posterior probability of each individual, the distance values of that individual were thereby withheld from estimation of conditional match and mismatch distributions in order to avoid overestimation of the posterior probability (This could inherently lead to non-monotonic posteriors (dependent on dist_xy_), but is negligible here). The prior matching probability P(match) is chosen as 0.001 assuming an a-priori chance of match being 1/1000. Practical example: Imagine a criminal case with 1000 potential suspects and only 1 saliva trace. The posterior probability for each person is the probability that the saliva trace stems from this person. As the posterior probability is here calculated for illustration purposes and the actual choice of the prior does not qualitatively change the resulting posterior probabilities, 1/1000 is considered a reasonable choice. The minimal match posterior is roughly 0.08 (Fig 4B), which is an increase in matching probability from 1/1000 to roughly 1/12.5. All other matching samples have higher posteriors, with 10 of 20 samples having matching probability of practically 100%. All estimated posteriors of mismatch samples decrease below the prior. For our practical example, this means that only for the person the saliva trace stems from, the probability increases to values higher than 1/1000, for all other suspects it decreases to values lower than 1/1000. The high change in probability from prior to posterior is essentially a consequence of low overlap of the estimated conditional probability distributions and results in high sensitivity and specificity of match and mismatch decisions. The variances of the match and mismatch distributions could potentially be further reduced by automatization and standardization of the whole assay, especially saliva sample preparation, and by using a higher number of antigens (peptide or protein) which are differentially reactive between individuals. However, already with 256 antigens a high individual matching probability is achieved due to low overlap of the estimated distributions. This indicates a high accordance of saliva and plasma IgG profiles.

## Discussion

Due to the relatively high heterogeneity of saliva across individuals as compared to plasma or blood (see Introduction), isolation of analytes from saliva is challenging. Still, we were able to develop a robust method for isolation of IgG from saliva (Fig 1) with high average purity (79% ± 13% SD). The procedure shows high robustness against various saliva compositions as we had samples from smokers and non-smokers, young and elderly individuals (24.4–56.5 years), as well as women and men. Average IgG yield of purified saliva samples was 5.3μg (final 0.15mg/mL in 35μL) from 2mL of saliva (S1 Table) which is roughly factor 2 lower than previously reported values [5]. The average amount of saliva required for our microarray experiments (2μg of purified IgG) was thus 0.75mL, the minimally required amount was 1.8mL for the sample with lowest concentration. The absolute IgG yield for each sample could potentially be enhanced by protocol optimization. However, our focus was high purity and high concentration of the isolated IgG antibodies. IgA content of purified saliva samples was below LOD of 1μg/mL. Although purification strategies for saliva and plasma are largely different (depletion and affinity purification, respectively), bands in gel electrophoresis and subsequent microarray analyses show highly similar profiles. This further emphasizes the suitability of the developed purification procedure.

We could show the diagnostic potential of saliva by implementing a procedure for anti-HBV antibody (HBsAg ad antigen) titer determination from saliva. The procedure was calibrated in order to infer plasma IgG titer levels from saliva IgG measurements. Titer values determined from saliva and plasma with our assay were compared to values determined by a reference lab from plasma (Fig 2). Concordance is reasonably high between reference values and our assay (R^2^ = 0.73). Comparing saliva and plasma reactivities (against HBsAg) of our assay reveals even higher concordance for paired samples (R^2^ = 0.91). This is in agreement with our initial hypothesis that IgG profiles in plasma and saliva are highly similar for each individual.

After showing high similarity of signal intensities between plasma and saliva for one HBV protein antigen, we generalised the result in a first step by performing correlation analyses on paired saliva and plasma samples of 20 individuals assayed on peptide microarrays (256 peptide antigens). The overall agreement of relative intensities in saliva and plasma is high (47% of the peptides show correlation coefficient r_xy_ > 0.8), see Fig 3. This indicates the general possibility of inferring relative and absolute plasma IgG reactivities from saliva IgG. In this way, standard plasma IgG based assays can, in principle, easily be adapted as saliva based assays by calibration, as shown in this work exemplarily for anti-HBV antibodies.

We showed that IgG profiles of plasma and saliva are highly similar for the 256 antigens used in our study (Fig 4 and S7 Fig). Although IgG antibodies against 256 antigens are only a small subset of the whole immunological repertoire of IgG antibodies, we suggest that still the results are generalizable. Before we continue with our argumentation we therefor need to address some general considerations about showing equality and inequality in a scientific/statistical sense in the context of immunological profiles.

Equality, inequality and statistical testing: It is in practice inherently hardly possible to define a scientific methodology for testing equality on continuous scales if no commonly accepted definitions of equality and inequality exist. This is i.e. the reason why the Null-hypothesis cannot be accepted in classical null hypothesis testing (e.g. T-test) if one fails to reject it. For continuous scales there are hardly situations where resulting values are exactly identical and thus the null hypothesis is strictly true, so we in principle anyway need bounds defining equality and inequality [27]. A possible solution to this could e.g. be that one tries to find conditional probability distributions of similarity measures for equality (match) and inequality (mismatch) for the specific problem. The resulting values can then be assigned to either distribution with a certain probability for a reasonable sample set/size. This approach is similar to a classical classification problem and one could thus calculate measures like sensitivity, specificity or ROC AUC. These values could further be tested on significance with various methods if desired [28–30].

In the context of the presented IgG profiling on peptide microarrays this means that we needed a procedure which takes all features (signal intensities of peptides and proteins) of two samples and calculates a similarity (or dissimilarity) measure between the two samples. We used 1 –Pearson’s r as distance metric. We then assigned a posterior probability of match or mismatch to each sample pair using the empirical conditional distribution and a pre-specified prior probability. All match probabilities increased to probabilities higher than the prior and all mismatch probabilities increased to probabilities lower than the prior. Note that choosing a different prior probability does not change the result qualitatively. Sensitivity and specificity of the according classical classification problem are each 100% or ROC AUC is 1. This can be considered a perfect result with respect to matching of plasma and saliva samples of each individual. The IgG profiles are unique for each person and are highly similar between saliva and plasma (at least for our study cohort and our measurement technology).

Definition of the immunological profile: The immunological profile of a person can be seen as a kind of immunological fingerprint. One could try to define the immunological profile e.g. as the complete immunological repertoire of a person by means of the concentrations of each antibody idiotype. However, the complete immunological (IgG) profile of a person is practically not measurable as the immunoglobulin repertoire (number of different idiotypes) in humans is at least 10^11^ and probably even several orders of magnitude greater [31,32]. Additionally, the immunological profile shows a temporal variation due to the dynamics of the immune system and changing environmental antigens (“The fingerprint changes over time.”). It is thus practically impossible to generate a complete immunological profile of a person with currently available technologies, at least to the best of our knowledge. Even the latest sequencing based technologies might not be capable of generating a full profile as not the sequence of the antibodies, but the final overall 3D structure (which is hard to predict) of the paratope is eventually defining the idiotype. As a consequence, one could anyway only generate an IgG profile on a subset of the immunological profile of a person and find good arguments which make generalisation of the results plausible.

For the presented analyses of IgG profiles and the resulting conclusions it is basically of minor relevance which particular subset of antigens (epitopes) is investigated or whether protein or peptide antigens are used, as long as there are at least a certain number of reactive antigens capable of discriminating between samples (otherwise technical noise would dominate the resulting data). Considering the thoughts above, there is yet no indication against generalisation of the results to the whole immunological profile, although we can only observe the profile partially. It is thus highly improbable that the analyses accidentally work only for the currently investigated subset, but not for any other subset. Or put another way, there is no indication that a certain subset of IgG idiotype gets into saliva and yet another subset does not. A more general “proof” of equality for plasma and saliva IgG profiles by means of an exhaustive analysis (e.g. with an antigen array covering “all possible antigens”) is currently unfeasible due to the above mentioned technical reasons.

We thus suggest generalisation of the results as: (i) One could anyway only observe a subset of the IgG profile due to the high number of possible different antibodies in the immunological repertoire; (ii) There is yet no indication that the results only hold for a specific subset of the IgG antibodies and that we accidentally picked one of these subsets.

Our hypothesis was that IgG profiles in plasma and saliva are highly similar for each individual. By means of consecutively showing high similarity of IgG reactivity profiles between plasma and saliva against multiple antigens we provide evidence for accepting this claim on a subset of the IgG profile and argue for generalisation to the entire IgG profile. The presented work thus provides a solid scientific basis for the development of saliva IgG based assays in the evolving field of saliva based diagnostics. With IgG being a rather large biomolecule, it is likely the case that many more molecules of diagnostic and prognostic interest, like hormones and metabolites, which are usually isolated from plasma are accessible through saliva. Although concentrations of most of the medically and scientifically interesting molecules in saliva are relatively low as compared to blood, we and others [3,18–21] could show that extraction and quantification of the molecules is feasible and practically utilisable. We emphasize that our study does neither provide a specific clinical test nor a specific medical application, but should be seen as an approach for showing that the IgG reactivities in saliva and plasma are generally highly similar for each individual. Nevertheless, the drawn conclusions provide a solid basis for numerous potential applications of saliva based diagnostics like e.g. (i) antibody titer determination for rapid verification of vaccination status; (ii) infection diagnostics and monitoring of epidemics or pandemics; (iii) testing of autoantibodies against tumour associated antigens or for diagnosis and monitoring of autoimmune disorders; (iv) monitoring of anti-rhesus antibodies in pregnancy and (v) testing of blood compatibility.

Practically the saliva based assays could potentially be implemented as lateral flow tests or microfluidic chips. The pore size of the device or an initial filter would thereby be designed in a way that mucins and other heavy components of the saliva sample are restrained, while antibodies (or other molecules of interest) can pass and subsequently are enriched on a capture platform. Detection and quantification can thereby be done by means of an indirect immunoassay with any enzyme or label based detection system like electrochemical or colorimetric detection. For non-enzymatic assays the test strips could potentially even be dried after sample application. In this way large numbers of samples could be collected and analysed conveniently and at low cost.

## Methods

### Experimental design

The goal of this study was to test our hypothesis that IgG profiles in plasma and saliva are highly similar for each individual. Confirming this hypothesis eventually leads to the conclusion that any plasma IgG based assay can, in principle, easily be adapted as saliva based assay and demonstrates the great potential of saliva based medical diagnostics. This was a study on voluntary healthy individuals and all subjects consented to participate in the study. No samples were excluded. All samples were de-identified except for sex and smoking status. Plasma and Saliva IgG samples were analysed on protein and peptide microarrays. Additionally, plasma samples were sent to a routine medical diagnostics lab for anti-HBV antibody titer determination.

### Samples

Peptide microarray: paired plasma and saliva samples of 20 individuals. Mean age 34.7 ± SD 8.7 (range 24.4–56.5) years. 8 smokers, 12 non-smokers. 12 males, 8 females.

HbsAg protein microarray: Paired plasma and saliva samples of 21 individuals. Mean age 35.4 ± 9.5 (27–58) years. 7 smokers, 14 non-smokers. 14 males, 7 females. Plasma samples of additional 17 individuals. Mean age 30.5 + 9.1 (20–48) years. 2 smokers, 15 non-smokers. 9 males, 8 females.

IDs of individuals are consistent throughout all figures and tables of the document. This study was approved by the local ethics committee of the city of Vienna (Magistratsabteilung 15 –Gesundheitsdienst der Stadt Wien, Ethikkommission der Stadt Wien). All participants provided verbal informed consent to participate in the study. All experiments were performed in accordance with relevant guidelines and regulations.

### Plasma and saliva sampling

Paired plasma and saliva samples were collected from healthy individuals simultaneously. All individuals had no known diseases by the time of sampling and 2 week afterwards. Saliva was collected according to the manufacturer’s instructions using a commercially available saliva collection system (Greiner 881002). In the course of collection the saliva is diluted roughly 1:1 with 4mL of 39mM citrate extraction buffer (see manufacturer’s instructions) and stored on ice. The collection procedure thus yields roughly 8mL of 1:1 saliva/buffer mixture, where 4mL of the mixture is used for further processing (see below). Within 30min after collection, the saliva/buffer mixture was immediately centrifuged at 2,200xg for 10min at room temperature (RT). The supernatant was micro-filtrated with 5μm and 0.45μm syringe filters (Merck SLSV025LS and SLHVM25NS) to remove mucous components and subsequently the filtrate was frozen at -20°C. EDTA plasma was prepared according to the manufacturer’s instructions immediately after collection (Greiner 456023) and aliquots were frozen at -20°C.

### Saliva IgG purification

Frozen aliquots of saliva/buffer mixture were thawed at room temperature. 4mL of saliva/buffer mixture was buffer exchanged in binding buffer (100mM sodium phosphate 150mM NaCl pH 7.2) and concentrated to 200μL using an Amicon 30k centrifugal filter device (Merck UFC803024). IgG was purified from this concentrate using a 96-well format Protein G column kit (Thermo 45204) according to the kit manual. In brief, the columns were pre-rinsed with binding buffer, 200μL of buffer exchanged sample was applied to the columns and incubated for 30min with moderate agitation at RT. Subsequently, columns were washed 4 times with binding buffer, bound IgG was eluted with 400μL of 100mM glycine pH 2.5 and pH was adjusted to 7.2 with 1M sodium phosphate pH 9. The eluate was buffer exchanged with deionized H_2_O and concentrated to 35μL using a Microcon 10k centrifugal filter device (Merck MRCPRT010) yielding a final concentration of roughly 0.15μg/μL or a total of 5.3μg IgG. Protein concentration was measured by spectrophotometry (Biotek Epoch Microplate Spectrophotometer with Take 3 module). Purity of IgG isolated from saliva was evaluated by SDS page as described below and by quantification of IgA on an in-house developed Luminex IgA quantification assay. The Luminex IgA quantification assay is based on a sandwich immunoassay. Capture antibody: Mouse anti-Human IgA Secondary Antibody (Thermo SA1-19258). Detection Antibody: Biotin-SP (long spacer) AffiniPure F(ab')₂ Fragment Goat Anti-Human Serum IgA, α Chain Specific (Jackson Immuno 109-066-011). Streptavidin-PE (Jackson Immuno 016-110-084) was used for detection and signals were read on a Luminex Flexmap 3D System. Samples were diluted in PBS with 1% BSA and PBS with 0.05% Tween-20 (Sigma, P9416) was used as wash buffer. The assay was calibrated with IgA standard (Sigma i4036) at concentrations 0, 1, 2.1, 4.2, 8.3, 16.6 μg/mL. For all assayed samples (n = 4), IgA signals were below limit of detection (LOD) of 1μg/mL.

### Plasma IgG purification

Plasma IgG was purified using a 96-well format Melon Gel kit (Thermo 45208) according to the kit manual with a slightly modified protocol. In brief, 15μL of plasma were diluted in 95μL of purification buffer, applied to the resin, incubated for 5 min with moderate agitation at RT and centrifuged at 1000g for 1min to obtain the purified IgG (roughly 1mg/mL in 100μL purification buffer). Purity was evaluated by SDS page.

### SDS page

SDS page was run with slight overloading of lanes to enhance visibility of impurities. 2.5μg of purified IgG (plasma and saliva) were applied to each well of precast polyacrylamide 4–12% gradient gels (Thermo NP0329BOX). Gels were run in 1x MOPS buffer (Thermo NP0001) with non-reducing conditions. Additionally, Pageruler Plus protein ladder (Thermo 26619) and human IgG standard (Sigma i2511) were run on two lanes. Gel was imaged and analysed using Chemidoc Touch with Imagelab 5.2.1 (Biorad).

### Peptide microarrays

Two different commercial peptide microarrays (JPT Berlin, Germany) mapping linear peptide epitopes of *Epstein-Barr virus* (EBV) nuclear antigen (EBNA, Swiss-Prot ID P03211) and *Hepatitis B virus* large envelope protein (HBV large envelope protein, Swiss-Prot ID P17101) were used. The EBV array consists of 158 peptides spotted in triplicates (RT-MW-EBV-EBNA1, JPT Berlin), the HBV array consists of 98 peptides spotted in triplicates (RT-MW-HBV-LEP, JPT Berlin) resulting in 256 analysed peptides. The linear peptide epitopes were derived as peptide scan from the protein antigens in terms of 15-mer peptides with 11 amino acids overlap.

### HBsAg protein microarray

*Hepatitis B virus* Surface Antigen ad (HBsAg ad, Biorad PIP002) was printed on standard format 2D Epoxy microarray slides (PolyAn 104 00 221) using an InkJet printing system (ArrayJet Marathon Argus). HBsAg was prepared at a concentration of 0.25mg/mL in 25mM phosphate buffer pH 8 + 0.02% SDS and subsequently mixed 1:1 (v/v) with pure ethylene glycol to increase viscosity for InkJet printing (60% relative humidity at RT). Slides were kept at 60% relative humidity and RT during printing and for 2 hours afterwards. Printed slides were stored at 4°C under vacuum. An international Anti-Hepatitis-B surface antigen (Anti-HBs) immunoglobulin standard (07/164, NIBSC, South Mimms, UK) was used for assay calibration. The Anti-HBs standard series for assay calibration was prepared in blocking buffer (PBS pH 7.4, 3% skimmed milk powder, 0.1% Tween-20) and processed like other samples (see Microarray processing).

### Reference anti-HBsAg antibody titer values

Plasma samples were sent to a routine medical diagnostic lab (Labors.at, Vienna, Austria) for determination of reference values for anti-HBsAg antibody titer (mIU/mL). The reference lab used Elecsys Anti-HBs II assay (Roche 05894816) on Roche cobas 6000 (e601 module).

### Microarray processing

Processing of microarrays was done equally for plasma and saliva IgG samples. Arrays were blocked with blocking buffer (PBS pH 7.4, 3% skimmed milk powder, 0.1% Tween-20) for 30 min with agitation (150rpm on plate shaker) at RT and washed three times with PBST (PBS pH 7.4, 0.1% Tween-20). Slides were spin-dried and incubated with 100μL of 0.02μg/μL sample IgG (2μg total IgG) in blocking buffer with agitation (150rpm) for 1.5 hours at RT. After washing for three times with PBST, slides were incubated with goat anti-human IgG Alexa 647 detection antibody (Invitrogen A21445) diluted 1:10,000 in blocking buffer with agitation for 1 hour at RT in the dark. Slides were washed another three times with PBST, two times with H2O and spin-dried immediately after the final wash step. Fluorescence intensity in the red channel (excitation laser 635nm, emission filter 676/37nm) was scanned with a Tecan PowerScanner.

### Microarray data

Fluorescence images of microarrays were processed with GenePix Pro 6.0 (Molecular devices) to quantify median fluorescence intensity (MFI), local background intensity (BG) and to identify low quality or damaged spots. No background correction was performed. Data was median-normalised to log2 MFI of 9 (or MFI = 512) for EBV and HBV arrays. Spots were removed from analyses if either: spot was flagged (flag = -100) or spot diameter was lower than 41 or higher than 150 (EBV) or 180 (HBV). Duplicate or triplicate spots were averaged. Then (averaged) data of EBV and HBV arrays were combined and normalised by adjusting the global median intensity of all HBV arrays to the global median intensity of all EBV arrays. EBV and HBV microarray data is available in the gene expression omnibus (GEO) repository [33] with accession number GSE117142. EBV and HBV array data can be accessed separately with accession numbers GSE117140 and GSE117141, respectively. HBsAg protein array data is provided in the supplement (S2 Table). HBsAg protein array data was not background corrected or normalized.

### Statistical analysis

Analyses were performed with R 3.4 [34] with packages limma and heatmap3 [35,36]. Weighted linear least squares regression was used for assay calibration with weights 1/titer and 1/titer_min_ for blanks. Predictions below 0 or larger than 1000 were set to 0 and 1000, respectively. Pearson’s r was used for calculation of correlation coefficients.

Hierarchical clustering was performed with distance function: dist_xy_ = 1 –r_xy_, r_xy_ is Pearson’s r. Single linkage was used as agglomeration method.

## Supporting information

S1 Fig IgG isolation from saliva and plasma with high purity. Relative chromatogram area under the curve (AUC) of plasma IgG and saliva IgG SDS page lanes is shown. The bars show the relative fraction of 100-250kDa bands on the total chromatogram AUC for the gel shown in (Fig 1A). With the IgG Standard (Std) as reference, average Saliva IgG purity is 79% (± 13% SD) and average Plasma IgG purity is 91% (± 9% SD).
(DOCX)

S2 Fig SDS page of concentrated saliva and purified saliva IgG. Lanes 1–8 show micro-filtrated (5μm and 0.45μm) and concentrated saliva (~ 8μg total protein). The Std lane shows the protein ladder (10, 15, 25, 35, 55, 70, 100, 130 and 250kDa). The IgG std is a commercial human IgG standard (2μg). Lanes 11–16 show IgG purified from saliva (~ 2.5μg total protein). Lane 17 shows BSA (1μg total protein).
(TIF)

S3 Fig Peptides showing low correlation between plasma and saliva samples show low mean MFI and/or low variance. Scatterplots of correlation values (r_xy_) between saliva IgG and plasma IgG samples against mean (left) and variance (right) of log MFI for 256 peptides (EBV and HBV). These diagrams indicate that peptides that are either low in mean reactivity or vary little between samples (weak differentiation power) show low correlation values.
(TIF)

S4 Fig Mean signal intensities and ranges of signal intensities are highly similar in plasma and saliva. Means and ranges of log2 MFI for 256 peptides (EBV and HBV) across 20 paired saliva IgG and plasma IgG samples are shown. This indicates a high concordance of relative IgG reactivities in saliva and plasma samples.
(TIF)

S5 Fig Paired saliva and plasma samples show high similarity of IgG profiles. Paired samples are more similar to each other than to saliva or plasma samples of any other individual. Pairwise scatterplots of log2 MFI signal intensities (256 peptides) for four representative paired plasma and saliva IgG samples. Correlation values are shown in white for each pair. Red solid lines are respective diagonals (x = y). Color represents correlation values with (red = 0.79 and blue = 1). Mean match correlation (saliva and plasma samples from same individual) is 0.963, mean mismatch correlation is 0.795 (Fig 4).
(TIF)

S6 Fig Pairwise clustering of saliva and plasma samples for all individuals. Heatmap of log2 MFI for saliva IgG and plasma IgG peptide microarrays is shown. Data was normalized according to the Materials and Methods part. Top color bars (red, blue) indicate saliva and plasma samples, respectively. Left color bar indicates peptides derived from EBV or HBV, as well as blank and control spots. Log2 MFI values of 20 paired samples are shown for 256 peptides and control peptides (process control peptides and blank spots).
(TIF)

S7 Fig Sample replicates and technical replicates show high similarity for an individual even when sampled more than six weeks separated. Hierarchical clustering of plasma IgG and saliva IgG samples analysed on EBV peptide microarrays. Technical replicates and sample replicates are included, see S4 Table for a more detailed description. The grey line indicates the highest dissimilarity of samples from one individual. Single linkage was used as agglomeration method and (1 –Pearson’s r) was used as distance. Terminal edges are colored in red.
(TIF)

S1 Table Concentrations and total amounts of IgG isolated from paired saliva and plasma samples. Final volume of saliva IgG isolate was 35μL, final volume of plasma IgG isolate was 100μL. Average IgG yield was 5.3μg (range 1.7–12.5μg) from 2mL of saliva and 54.8μg (range 40–76.45μg) from 15μL of plasma.
(DOCX)

S2 Table Signal intensities and titer values for plasma and saliva samples. MFI, reference titer values (anti-HBs titer) and predicted titer values for IgG samples isolated from saliva and plasma. Paired plasma and saliva samples were available from 21 individuals, plasma samples were available from additional 17 individuals. Reference titer values for all individuals were measured in plasma samples (see Materials and Methods part). Predicted titer values (mIU/mL) were calculated with the regression formula from S3 Table.
(DOCX)

S3 Table Calibration of the anti-HBV titer assay. The spotted HBsAg (ad) protein microarray was calibrated with an anti-HBs antibody standard obtained from NIBSC. The calibration equation is: MFI = 204.76 + 3.108 * (anti-HBs titer). Weighted linear least squares regression was used for data fitting (see Materials and Methods part).
(DOCX)

S4 Table Specification of samples analysed on the EBV peptide microarray. Technical replicates (repeated IgG purification and microarray processing from the same sample) and sample replicates (repeated sampling from one individual) are included and indicated with a dot and a consecutive number after the individual number (1–20) and sample type (plasma P and saliva S). Samples 1.Pla.4, 1.Sal.2, 14.Sal.2, 14.Sal.4, 17.Pla.2, 2.Pla.3 and 2.Sal.2 are technical replicates of the according previous sample ID (e.g. 1.Pla.4 is the technical replicate of 1.Pla.3).
(DOCX)
