# Supplementary material for: High similarity of IgG antibody profiles in blood and saliva opens opportunities for saliva based serology
Source: PLoS One. 2019 Jun 20;14(6):e0218456. doi: 10.1371/journal.pone.0218456 (PMC6586443; doi:10.1371/journal.pone.0218456)
Supplement: S1 Fig — Relative chromatogram area under the curve (AUC) of plasma IgG and saliva IgG SDS page lanes is shown. The bars show the relative fraction of 100-250kDa bands on the total chromatogram AUC for the gel shown in (Fig 1A). With the IgG Standard (Std) as reference, average Saliva IgG purity is 79% (± 13% SD) and average Plasma IgG purity is 91% (± 9% SD). (DOCX) [file pone.0218456.s001.docx]

**S1 Fig. IgG isolation from saliva and plasma with high purity.** Relative chromatogram area under the curve (AUC) of plasma IgG and saliva IgG SDS page lanes is shown. The bars show the relative fraction of 100-250kDa bands on the total chromatogram AUC for the gel shown in (Fig 1A). With the IgG Standard (Std) as reference, average Saliva IgG purity is 79% (± 13% SD) and average Plasma IgG purity is 91% (± 9% SD).
