# Supplementary figures and images for: High similarity of IgG antibody profiles in blood and saliva opens opportunities for saliva based serology
Source: PLoS One. 2019 Jun 20;14(6):e0218456. doi: 10.1371/journal.pone.0218456 (PMC6586443; doi:10.1371/journal.pone.0218456)

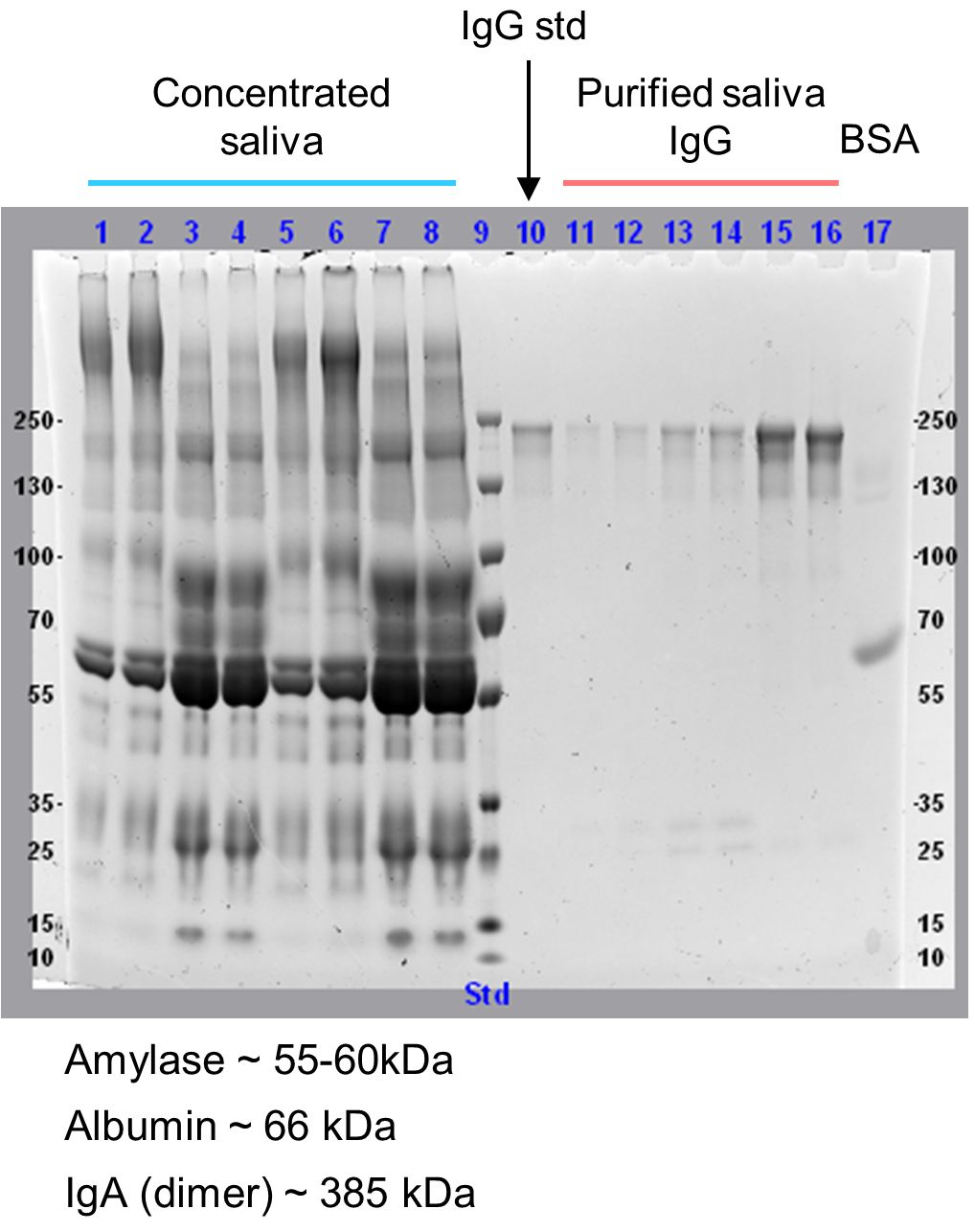

Supplement: S2 Fig — Lanes 1–8 show micro-filtrated (5μm and 0.45μm) and concentrated saliva (~ 8μg total protein). The Std lane shows the protein ladder (10, 15, 25, 35, 55, 70, 100, 130 and 250kDa). The IgG std is a commercial human IgG standard (2μg). Lanes 11–16 show IgG purified from saliva (~ 2.5μg total protein). Lane 17 shows BSA (1μg total protein). (TIF) [file pone.0218456.s002.tif]

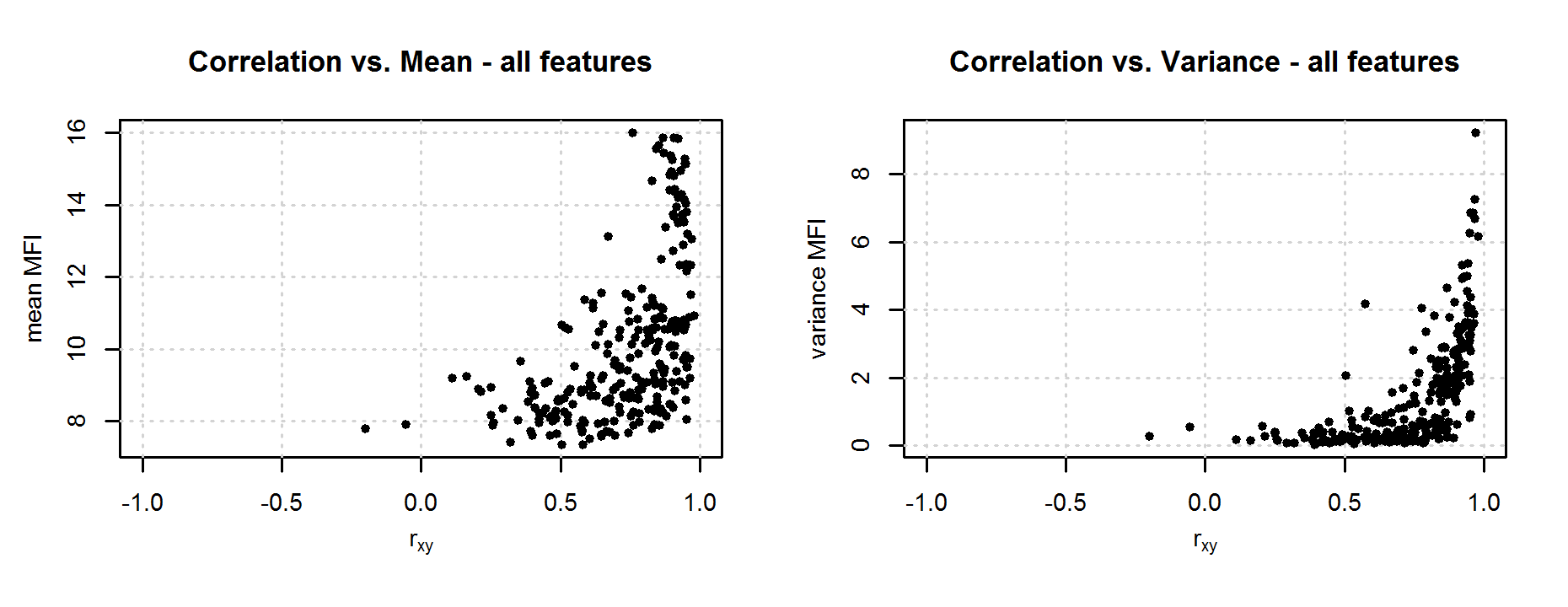

Supplement: S3 Fig — Scatterplots of correlation values (rxy) between saliva IgG and plasma IgG samples against mean (left) and variance (right) of log MFI for 256 peptides (EBV and HBV). These diagrams indicate that peptides that are either low in mean reactivity or vary little between samples (weak differentiation power) show low correlation values. (TIF) [file pone.0218456.s003.tif]

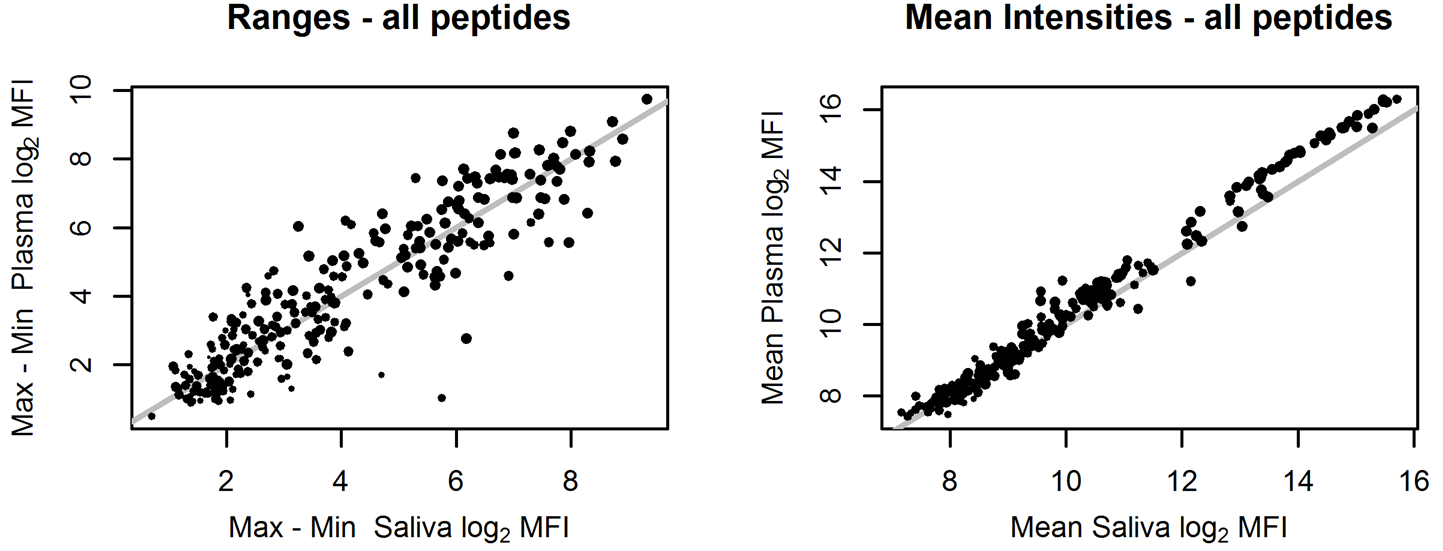

Supplement: S4 Fig — Means and ranges of log2 MFI for 256 peptides (EBV and HBV) across 20 paired saliva IgG and plasma IgG samples are shown. This indicates a high concordance of relative IgG reactivities in saliva and plasma samples. (TIF) [file pone.0218456.s004.tif]

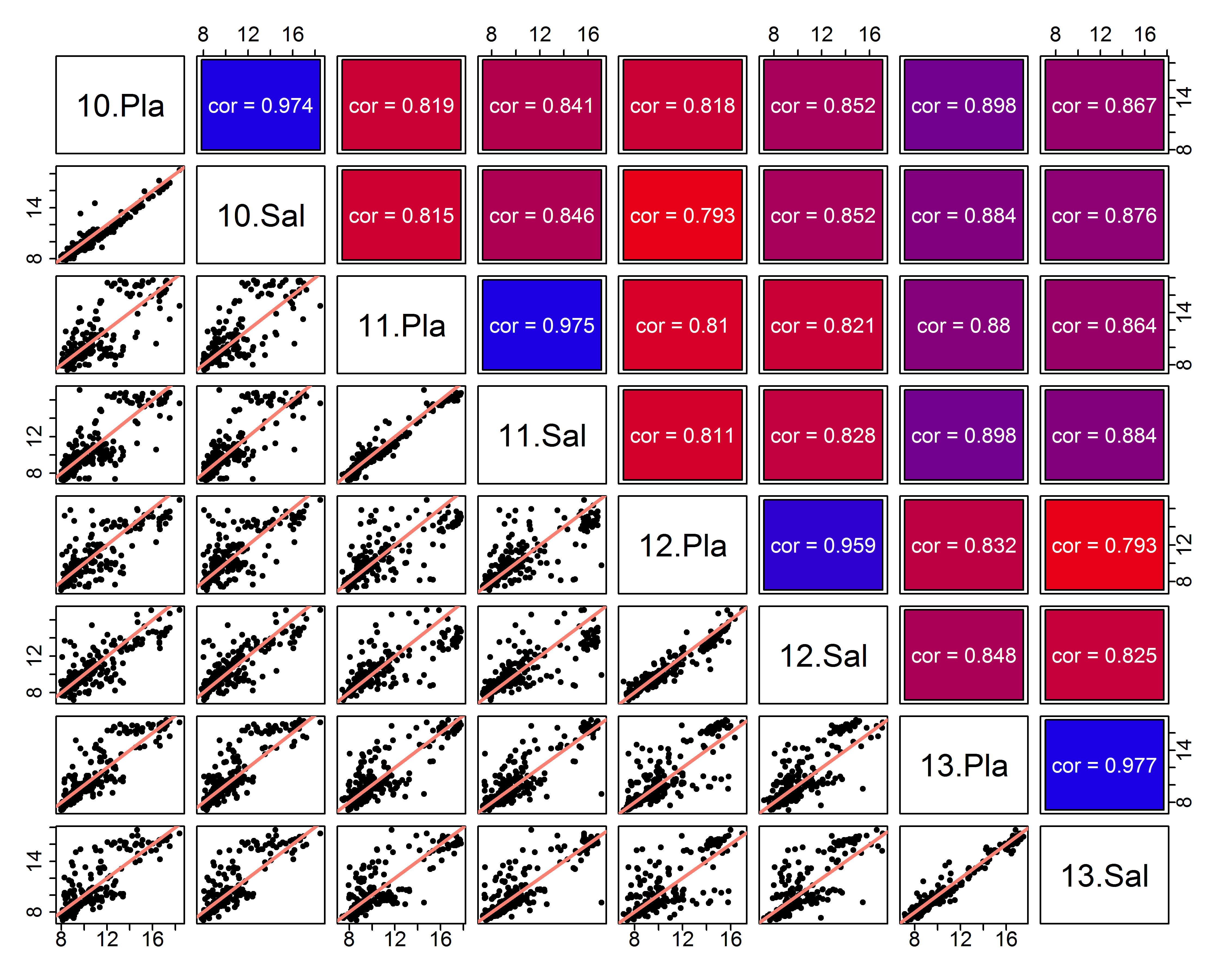

Supplement: S5 Fig — Paired samples are more similar to each other than to saliva or plasma samples of any other individual. Pairwise scatterplots of log2 MFI signal intensities (256 peptides) for four representative paired plasma and saliva IgG samples. Correlation values are shown in white for each pair. Red solid lines are respective diagonals (x = y). Color represents correlation values with (red = 0.79 and blue = 1). Mean match correlation (saliva and plasma samples from same individual) is 0.963, mean mismatch correlation is 0.795 (Fig 4). (TIF) [file pone.0218456.s005.tif]

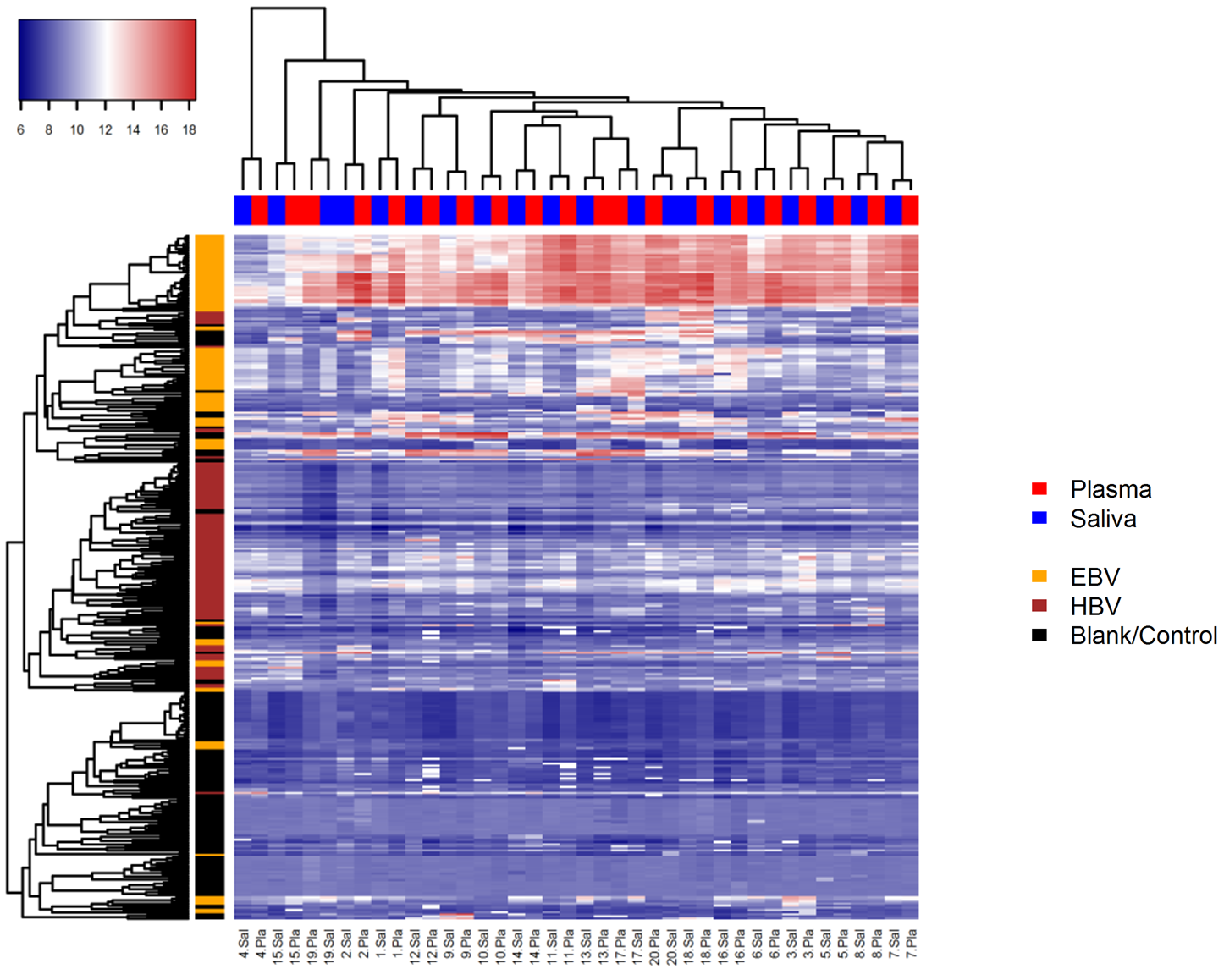

Supplement: S6 Fig — Heatmap of log2 MFI for saliva IgG and plasma IgG peptide microarrays is shown. Data was normalized according to the Materials and Methods part. Top color bars (red, blue) indicate saliva and plasma samples, respectively. Left color bar indicates peptides derived from EBV or HBV, as well as blank and control spots. Log2 MFI values of 20 paired samples are shown for 256 peptides and control peptides (process control peptides and blank spots). (TIF) [file pone.0218456.s006.tif]

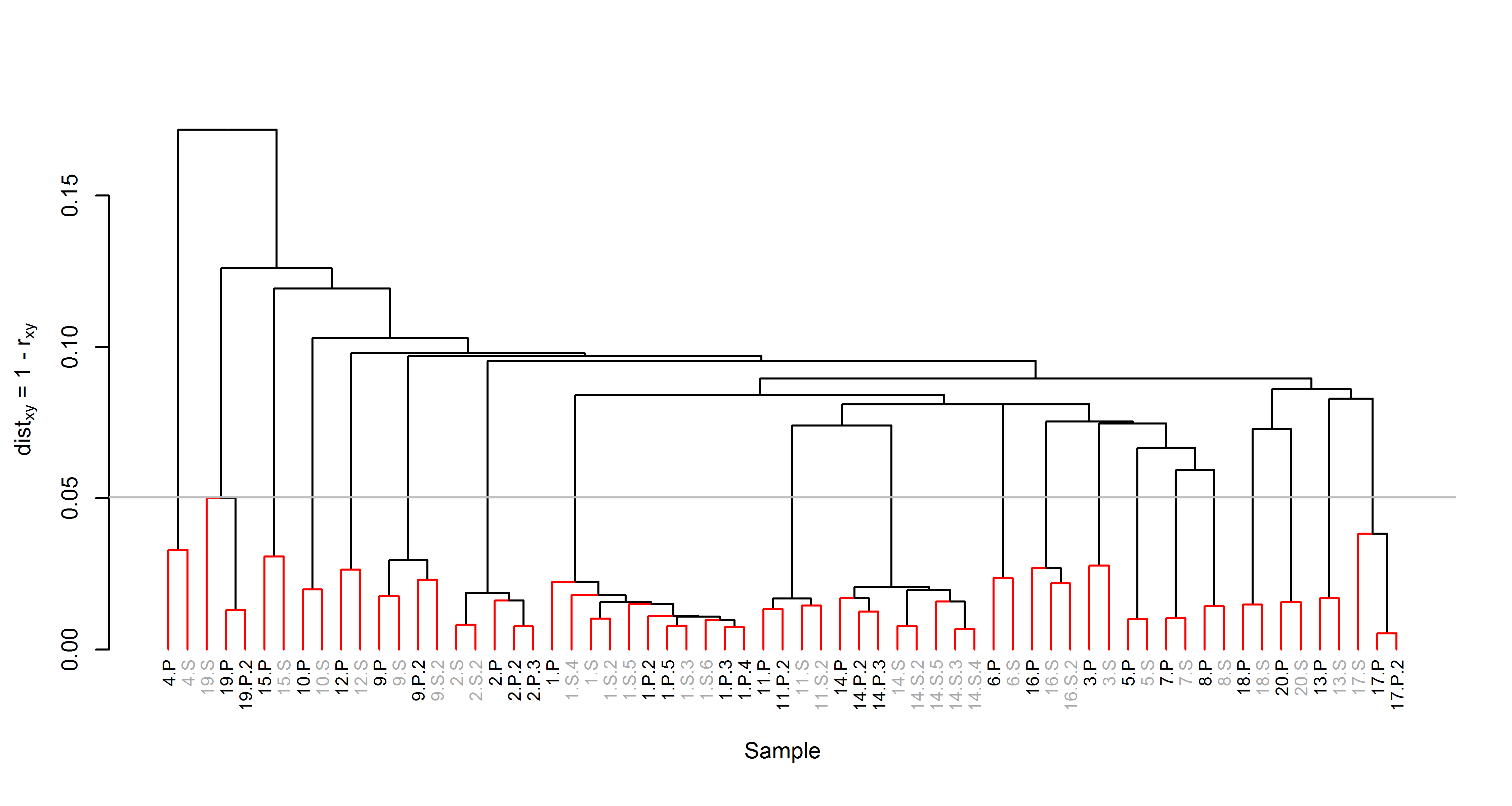

Supplement: S7 Fig — Hierarchical clustering of plasma IgG and saliva IgG samples analysed on EBV peptide microarrays. Technical replicates and sample replicates are included, see S4 Table for a more detailed description. The grey line indicates the highest dissimilarity of samples from one individual. Single linkage was used as agglomeration method and (1 –Pearson’s r) was used as distance. Terminal edges are colored in red. (TIF) [file pone.0218456.s007.tif]
