# Supplementary material for: High similarity of IgG antibody profiles in blood and saliva opens opportunities for saliva based serology
Source: PLoS One. 2019 Jun 20;14(6):e0218456. doi: 10.1371/journal.pone.0218456 (PMC6586443; doi:10.1371/journal.pone.0218456)
Supplement: S1 Table — Final volume of saliva IgG isolate was 35μL, final volume of plasma IgG isolate was 100μL. Average IgG yield was 5.3μg (range 1.7–12.5μg) from 2mL of saliva and 54.8μg (range 40–76.45μg) from 15μL of plasma. (DOCX) [file pone.0218456.s008.docx]

| **Individual** | **Final Saliva IgG conc (µg/µL)** | **Final Saliva IgG**  **(µg)** | **Final Plasma IgG**  **conc (µg/µL)** | **Final Plasma IgG**  **(µg)** |
| --- | --- | --- | --- | --- |
| **1** | 0.36 | 12.5 | 0.65 | 65.0 |
| **2** | 0.15 | 5.3 | 0.41 | 40.7 |
| **3** | 0.06 | 2.2 | 0.40 | 40.5 |
| **4** | 0.08 | 2.9 | 0.70 | 70.3 |
| **5** | 0.10 | 3.5 | 0.56 | 56.0 |
| **6** | 0.10 | 3.6 | 0.46 | 45.7 |
| **7** | 0.26 | 9.0 | 0.58 | 58.0 |
| **8** | 0.14 | 5.0 | 0.41 | 41.3 |
| **9** | 0.05 | 1.7 | 0.53 | 53.0 |
| **10** | 0.23 | 7.9 | 0.76 | 76.5 |
| **11** | 0.06 | 2.2 | 0.53 | 52.6 |
| **12** | 0.10 | 3.6 | 0.53 | 52.6 |
| **13** | 0.21 | 7.3 | 0.40 | 40.0 |
| **14** | 0.13 | 4.4 | 0.70 | 69.6 |
| **15** | 0.08 | 2.7 | 0.47 | 46.7 |
| **16** | 0.15 | 5.3 | 0.64 | 64.0 |
| **17** | 0.30 | 10.5 | 0.45 | 44.7 |
| **18** | 0.11 | 3.7 | 0.68 | 68.5 |
| **19** | 0.28 | 9.8 | 0.49 | 48.5 |
| **20** | 0.08 | 2.7 | 0.62 | 62.2 |

**S1 Table. Concentrations and total amounts of IgG isolated from paired saliva and plasma samples.** Final volume of saliva IgG isolate was 35µL, final volume of plasma IgG isolate was 100µL. Average IgG yield was 5.3µg (range 1.7 – 12.5µg) from 2mL of saliva and 54.8µg (range 40 – 76.45µg) from 15µL of plasma.
