# Supplementary material for: High similarity of IgG antibody profiles in blood and saliva opens opportunities for saliva based serology
Source: PLoS One. 2019 Jun 20;14(6):e0218456. doi: 10.1371/journal.pone.0218456 (PMC6586443; doi:10.1371/journal.pone.0218456)
Supplement: S2 Table — MFI, reference titer values (anti-HBs titer) and predicted titer values for IgG samples isolated from saliva and plasma. Paired plasma and saliva samples were available from 21 individuals, plasma samples were available from additional 17 individuals. Reference titer values for all individuals were measured in plasma samples (see Materials and Methods part). Predicted titer values (mIU/mL) were calculated with the regression formula from S3 Table. (DOCX) [file pone.0218456.s009.docx]

| **Individual** | **Saliva/Plasma**  **IgG** | **anti-HBs titer**  **(mIU/mL)** | **MFI** | **predicted**  **(mIU/mL)** |
| --- | --- | --- | --- | --- |
| **1** | Plasma | >1000 | 14971 | >1000 |
| **1** | Saliva | >1000 | 14340 | >1000 |
| **2** | Plasma | >1000 | 12250 | >1000 |
| **2** | Saliva | >1000 | 3114 | 936 |
| **3** | Plasma | 117 | 847 | 207 |
| **3** | Saliva | 117 | 429 | 72 |
| **4** | Plasma | 2 | 404 | 64 |
| **4** | Saliva | 2 | 322 | 38 |
| **5** | Plasma | 14 | 406 | 65 |
| **6** | Plasma | 235 | 2086 | 605 |
| **6** | Saliva | 235 | 2252 | 659 |
| **7** | Plasma | 229 | 1149 | 304 |
| **7** | Saliva | 229 | 742 | 173 |
| **8** | Plasma | >1000 | 2983 | 894 |
| **9** | Plasma | 542 | 2443 | 720 |
| **9** | Saliva | 542 | 1296 | 351 |
| **10** | Plasma | 121 | 471 | 86 |
| **11** | Plasma | 2 | 388 | 59 |
| **11** | Saliva | 2 | 360 | 50 |
| **12** | Plasma | 292 | 885 | 219 |
| **12** | Saliva | 292 | 963 | 244 |
| **13** | Plasma | 353 | 1328 | 361 |
| **13** | Saliva | 353 | 1290 | 349 |
| **14** | Plasma | 263 | 675 | 151 |
| **14** | Saliva | 263 | 621 | 134 |
| **15** | Plasma | >1000 | 2910 | 871 |
| **15** | Saliva | >1000 | 700 | 159 |
| **16** | Plasma | >1000 | 23548 | >1000 |
| **17** | Plasma | 689 | 2544 | 753 |
| **17** | Saliva | 689 | 1183 | 315 |
| **18** | Plasma | >1000 | 39465 | >1000 |
| **18** | Saliva | >1000 | 64609 | >1000 |
| **19** | Plasma | 2 | 883 | 218 |
| **19** | Saliva | 2 | 447 | 78 |
| **20** | Plasma | >1000 | 65535 | >1000 |
| **20** | Saliva | >1000 | 65535 | >1000 |
| **21** | Plasma | 89 | 404 | 64 |
| **21** | Saliva | 89 | 334 | 42 |
| **22** | Plasma | 273 | 1684 | 476 |
| **22** | Saliva | 273 | 831 | 201 |
| **23** | Plasma | >1000 | 1798 | 513 |
| **23** | Saliva | >1000 | 813 | 196 |
| **24** | Plasma | 0 | 494 | 93 |
| **24** | Saliva | 0 | 326 | 39 |
| **25** | Plasma | 119 | 490 | 92 |
| **25** | Saliva | 119 | 506 | 97 |
| **26** | Plasma | 287 | 533 | 106 |
| **27** | Plasma | 0 | 323 | 38 |
| **28** | Plasma | 350 | 822 | 199 |
| **29** | Plasma | 224 | 520 | 101 |
| **30** | Plasma | 68 | 402 | 64 |
| **31** | Plasma | 25 | 458 | 81 |
| **32** | Plasma | 182 | 414 | 67 |
| **33** | Plasma | >1000 | 2015 | 583 |
| **34** | Plasma | 0 | 345 | 45 |
| **35** | Plasma | >1000 | 5400 | >1000 |
| **36** | Plasma | 645 | 2163 | 630 |
| **37** | Plasma | 249 | 628 | 136 |
| **38** | Plasma | 19 | 305 | 32 |

**S2 Table. Signal intensities and titer values for plasma and saliva samples.** MFI, reference titer values (anti-HBs titer) and predicted titer values for IgG samples isolated from saliva and plasma. Paired plasma and saliva samples were available from 21 individuals, plasma samples were available from additional 17 individuals. Reference titer values for all individuals were measured in plasma samples (see Materials and Methods part). Predicted titer values (mIU/mL) were calculated with the regression formula from S3 Table.
