# Supplementary material for: High similarity of IgG antibody profiles in blood and saliva opens opportunities for saliva based serology
Source: PLoS One. 2019 Jun 20;14(6):e0218456. doi: 10.1371/journal.pone.0218456 (PMC6586443; doi:10.1371/journal.pone.0218456)
Supplement: S3 Table — The spotted HBsAg (ad) protein microarray was calibrated with an anti-HBs antibody standard obtained from NIBSC. The calibration equation is: MFI = 204.76 + 3.108 * (anti-HBs titer). Weighted linear least squares regression was used for data fitting (see Materials and Methods part). (DOCX) [file pone.0218456.s010.docx]

| **anti-HBs titer (mIU/mL)** | **MFI** |
| --- | --- |
| **1000.0** | 3729 |
| **500.0** | 1355 |
| **250.0** | 702 |
| **125.0** | 739 |
| **62.5** | 480 |
| **31.3** | 336 |
| **15.6** | 235 |
| **7.8** | 222 |
| **3.9** | 248 |
| **0** | 201 |
| **0** | 195 |
| **0** | 187 |

**S3 Table. Calibration of the anti-HBV titer assay.** The spotted HBsAg (ad) protein microarray was calibrated with an anti-HBs antibody standard obtained from NIBSC. The calibration equation is: MFI = 204.76 + 3.108 * (anti-HBs titer). Weighted linear least squares regression was used for data fitting (see Materials and Methods part).
