# Supplementary material for: High similarity of IgG antibody profiles in blood and saliva opens opportunities for saliva based serology
Source: PLoS One. 2019 Jun 20;14(6):e0218456. doi: 10.1371/journal.pone.0218456 (PMC6586443; doi:10.1371/journal.pone.0218456)
Supplement: S4 Table — Technical replicates (repeated IgG purification and microarray processing from the same sample) and sample replicates (repeated sampling from one individual) are included and indicated with a dot and a consecutive number after the individual number (1–20) and sample type (plasma P and saliva S). Samples 1.Pla.4, 1.Sal.2, 14.Sal.2, 14.Sal.4, 17.Pla.2, 2.Pla.3 and 2.Sal.2 are technical replicates of the according previous sample ID (e.g. 1.Pla.4 is the technical replicate of 1.Pla.3). (DOCX) [file pone.0218456.s011.docx]

| **Individual** | **Plasma/Saliva** | **Day of sampling**  **and processing** | **ID** | **Gender** | **Smoker** | **Comment** |
| --- | --- | --- | --- | --- | --- | --- |
| **1** | Plasma | 0 | 1.Pla | m | n |  |
| **1** | Plasma | 0 | 1.Pla.2 | m | n |  |
| **1** | Plasma | -43 | 1.Pla.3 | m | n |  |
| **1** | Plasma | -43 | 1.Pla.4 | m | n | technical replicate |
| **1** | Plasma | 0 | 1.Pla.5 | m | n |  |
| **1** | Saliva | -43 | 1.Sal | m | n |  |
| **1** | Saliva | -43 | 1.Sal.2 | m | n | technical replicate |
| **1** | Saliva | 0 | 1.Sal.3 | m | n |  |
| **1** | Saliva | 0 | 1.Sal.4 | m | n |  |
| **1** | Saliva | 0 | 1.Sal.5 | m | n |  |
| **1** | Saliva | 0 | 1.Sal.6 | m | n |  |
| **2** | Plasma | 0 | 2.Pla | f | n |  |
| **2** | Plasma | -43 | 2.Pla.2 | f | n |  |
| **2** | Plasma | -43 | 2.Pla.3 | f | n | technical replicate |
| **2** | Saliva | -43 | 2.Sal | f | n |  |
| **2** | Saliva | -43 | 2.Sal.2 | f | n | technical replicate |
| **3** | Plasma | 0 | 3.Pla | f | n |  |
| **3** | Saliva | 0 | 3.Sal | f | n |  |
| **4** | Plasma | 0 | 4.Pla | m | n |  |
| **4** | Saliva | 0 | 4.Sal | m | n |  |
| **5** | Plasma | 0 | 5.Pla | m | n |  |
| **5** | Saliva | 0 | 5.Sal | m | n |  |
| **6** | Plasma | 0 | 6.Pla | f | n |  |
| **6** | Saliva | 0 | 6.Sal | f | n |  |
| **7** | Plasma | 0 | 7.Pla | f | n |  |
| **7** | Saliva | 0 | 7.Sal | f | n |  |
| **8** | Plasma | 0 | 8.Pla | f | y |  |
| **8** | Saliva | 0 | 8.Sal | f | y |  |
| **9** | Plasma | 0 | 9.Pla | m | n |  |
| **9** | Plasma | -38 | 9.Pla.2 | m | n |  |
| **9** | Saliva | 0 | 9.Sal | m | n |  |
| **9** | Saliva | -38 | 9.Sal.2 | m | n |  |
| **10** | Plasma | 0 | 10.Pla | m | n |  |
| **10** | Saliva | 0 | 10.Sal | m | n |  |
| **11** | Plasma | 0 | 11.Pla | m | y |  |
| **11** | Plasma | -38 | 11.Pla.2 | m | y |  |
| **11** | Saliva | 0 | 11.Sal | m | y |  |
| **11** | Saliva | -38 | 11.Sal.2 | m | y |  |
| **12** | Plasma | 0 | 12.Pla | m | n |  |
| **12** | Saliva | 0 | 12.Sal | m | n |  |
| **13** | Plasma | 0 | 13.Pla | m | n |  |
| **13** | Saliva | 0 | 13.Sal | m | n |  |
| **14** | Plasma | 0 | 14.Pla | m | y |  |
| **14** | Plasma | -38 | 14.Pla.2 | m | y |  |
| **14** | Plasma | 0 | 14.Pla.3 | m | y |  |
| **14** | Saliva | 0 | 14.Sal | m | y |  |
| **14** | Saliva | 0 | 14.Sal.2 | m | y | technical replicate |
| **14** | Saliva | 0 | 14.Sal.3 | m | y |  |
| **14** | Saliva | 0 | 14.Sal.4 | m | y | technical replicate |
| **14** | Saliva | -38 | 14.Sal.5 | m | y |  |
| **15** | Plasma | 0 | 15.Pla | m | y |  |
| **15** | Saliva | 0 | 15.Sal | m | y |  |
| **16** | Plasma | 0 | 16.Pla | f | n |  |
| **16** | Saliva | 0 | 16.Sal | f | n |  |
| **16** | Saliva | 0 | 16.Sal.2 | f | n |  |
| **17** | Plasma | 0 | 17.Pla | m | y |  |
| **17** | Plasma | 0 | 17.Pla.2 | m | y | technical replicate |
| **17** | Saliva | 0 | 17.Sal | m | y |  |
| **18** | Plasma | 0 | 18.Pla | f | y |  |
| **18** | Saliva | 0 | 18.Sal | f | y |  |
| **19** | Plasma | 0 | 19.Pla | f | n |  |
| **19** | Plasma | 0 | 19.Pla.2 | f | n |  |
| **19** | Saliva | 0 | 19.Sal | f | n |  |
| **20** | Plasma | 0 | 20.Pla | m | y |  |
| **20** | Saliva | 0 | 20.Sal | m | y |  |

**S4 Table. Specification of samples analysed on the EBV peptide microarray.** Technical replicates (repeated IgG purification and microarray processing from the same sample) and sample replicates (repeated sampling from one individual) are included and indicated with a dot and a consecutive number after the individual number (1-20) and sample type (plasma P and saliva S). Samples 1.Pla.4, 1.Sal.2, 14.Sal.2, 14.Sal.4, 17.Pla.2, 2.Pla.3 and 2.Sal.2 are technical replicates of the according previous sample ID (e.g. 1.Pla.4 is the technical replicate of 1.Pla.3).
